# Local References for Ultrasound-Estimated Fetal Weight Based on 2,211 Singleton Pregnancies in the City of Curitiba, South of Brazil

**DOI:** 10.1055/s-0040-1709691

**Published:** 2020-04

**Authors:** Daniel Massamatsu Pianovski Kato, Liziane Lorusso, Natália Roberta Andrade Dalla Costa, Camila Rotter Queiroz Ulyssea, Gabrielle Navarro Lizana, Luana Lenzi, Rafael Frederico Bruns, Elisa Chicareli Pinhat, Edward Araujo Júnior

**Affiliations:** 1 Department of Obstetrics and Gynecology, Universidade Federal do Paraná, Curitiba, PR, Brazil; 2 Department of Gynecology and Obstetrics, Universidade Positivo, Curitiba, PR, Brazil; 3 Department of Obstetrics, Escola Paulista de Medicina, Universidade Federal de São Paulo, São Paulo, SP, Brazil; 4 Medical Course, Universidade Municipal de São Caetano do Sul, São Paulo, SP, Brazil

**Keywords:** fetal weight, prenatal ultrasonography, growth charts, percentiles, reference curves, peso fetal, ultrassonografia pré-natal, curvas de crescimento, percentis, curvas de referência

## Abstract

**Objective** To develop reference curves of estimated fetal weight for a local population in Curitiba, South of Brazil, and compare them with the curves established for other populations.

**Methods** An observational, cross-sectional, retrospective study was conducted. A reference model for estimated fetal weight was developed using a local sample of 2,211 singleton pregnancies with low risk of growth disorders and well-defined gestational age. This model was compared graphically with the Hadlock and Intergrowth 21^st^ curves.

**Results** Reference curves for estimated fetal weight were developed for a local population. The coefficient of determination was R^2^ = 99.11%, indicating that 99.11% of the fetal weight variations were explained by the model. Compared with Hadlock curves, the 50^th^, 90^th^, and 97^th^ percentiles in this model were lower, whereas the 10^th^ percentile nearly overlapped, and the 3^rd^ percentile was slightly higher in the proposed model. The percentiles were higher in the proposed model compared with the Intergrowth 21^st^ curves, particularly for the 3^rd^, 10^th^, and 50^th^ percentiles.

**Conclusion** We provide a local reference curve for estimated fetal weight. The proposed model was different from other models, and these differences might be due to the use of different populations for model construction.

## Resumo

**Objetivo** Desenvolver curvas de referência para o peso fetal estimado em uma população de Curitiba, Sul do Brasil, e compará-las com curvas estabelecidas para outras populações.

**Métodos** Foi realizado um estudo observacional, transversal e retrospectivo. Um modelo de referência para o peso fetal estimado foi desenvolvido usando uma amostra local de 2.211 gestações únicas de baixo risco de distúrbios do crescimento e idade gestacional bem definida. Este modelo foi comparado graficamente com as curvas de Hadlock e *Intergrowth 21* ^st^.

**Resultados** As curvas de referência para o peso fetal estimado foram desenvolvidas para uma população local. O coeficiente de determinação foi de R^2^ = 99,11%, indicando que 99,11% das variações do peso fetal foram explicadas pelo modelo. Em comparação com as curvas de Hadlock, os percentis 50, 90, e 97 neste modelo foram inferiores, enquanto o percentil 10 quase se sobrepôs, e o percentil 3 foi ligeiramente superior no modelo proposto. Os percentis foram maiores no modelo proposto em comparação com as curvas do *Intergrowth 21st*, particularmente para os percentis 3, 10, e 50.

**Conclusão** Fornecemos uma curva de referência local para o peso fetal estimado. O modelo proposto foi diferente de outros modelos, e essas diferenças podem ser devido ao uso de diferentes populações para a construção do modelo.

## Introduction

Changes in intrauterine growth and prematurity are the major determinants of neonatal morbidity and mortality.^1^ Intrauterine growth restriction (IUGR), a condition in which a fetus cannot reach its biological growth potential,^2^ is strongly associated with perinatal morbidity and mortality and acute fetal distress. It is estimated that 1 to 2% of neonatal deaths worldwide are a direct result of IUGR in term newborns.^3^ In addition, there is a correlation between IUGR and delayed neurodevelopment^4^ and chronic diseases in adulthood, including chronic arterial hypertension, type II diabetes mellitus, and cardiovascular diseases.^5^ Macrosomic or large-for-gestational-age fetuses present higher risk of intrauterine death and adverse perinatal outcomes, such as shoulder dystocia, humeral and clavicular fracture, brachial plexus and facial palsy, asphyxia, meconium aspiration, hypoglycemia, neonatal hyperbilirubinemia, hypertrophic cardiomyopathy, and prolonged stay in intensive care units.^6^ Therefore, the adequate identification of these fetuses is crucial for prenatal management and for determining the optimal time for delivery.

Some studies have provided reference charts for fetal biometric parameters, although there are discrepancies in median values and percentile curves.^7^ ^8^ These differences may be due to racial, maternal, biological, and demographic factors^9^ as well as to methodological failure in published studies.^10^

The data published by the Brazilian Institute of Geography and Statistics in the 2010 census^11^ indicated that 47.7% of Brazilians are classified as white, 43.1% as mixed race, 7.6% as black, 1.05% as Asian, and 0.43% as indigenous people. Therefore, almost 50% of the Brazilian population can be considered of mixed race. In the city of Curitiba, 78% of the population are white, 2.8% are black, 1.3% are Asian, and 16% are of mixed race,^12^ evidencing ethnic variations even within a single country. In Brazil, the most used reference is that published by Hadlock et al,^13^ which is based on a predominantly Caucasian and middle-class population comprising 392 pregnant women.

Based on this information, we developed a reference curve for the city of Curitiba, South of Brazil, using data obtained from 2,211 tests conducted in a population classified as having a low risk of fetal growth disorders.

## Methods

The present observational, cross-sectional and retrospective study was approved by the research ethics committee of the Hospital of Universidade Federal do Paraná (UFPR) on March 30, 2016 under Opinion No. 1.470.703.

For developing the estimated fetal weight reference curve, pregnant women at gestational weeks 14 to 41 were subjected to routine ultrasound examination at a private clinic in Curitiba from March 2011 to March 2015. The examinations were conducted by 10 medical specialists in fetal medicine using GE ultrasound devices models Voluson 730 Expert and Voluson S6 (GE Medical System, Zipf, Austria).

The inclusion criteria were low-risk pregnant women with a singleton gestation and well-defined gestational age, confirmed by the date of the last menstrual period, when the difference between this date and first trimester ultrasound was less than 5 days, or by the measurement of the crown-to-rump length (CRL) in an examination conducted before the gestational age of 13 weeks and 6 days. Multiple pregnancies, fetuses with congenital malformations or chromosomal abnormalities, congenital infections, fetal deaths, and pregnant women with diseases associated with fetal growth disorders (chronic arterial hypertension, gestational hypertension, preeclampsia, previous or gestational diabetes mellitus, chronic renal disease, chronic pulmonary disease, cyanotic heart disease, alcohol abuse, smoking, systemic lupus erythematosus, antiphospholipid syndrome, and thrombophilia)as well as those who were living in high altitudes were excluded.

A total of 8,447 examinations were conducted in 2,211 patients. Gestational age in weeks and days and fetal weight in grams (estimated by Hadlock's formula: Log 10 [weight] = 1.3596–[0. 00386 × AC × FL] + [0.0064 × HC] + [0.00061 × BPD × AC] + [0.0424 × AC] + [0.174 × FL], in which AC is abdominal circumference, FL is femoral length, HC is head circumference, and BPD is biparietal diameter) were recorded in a Microsoft Excel 2007 worksheet (Microsoft Corporation, Redmond, WA, USA).

For the inclusion of only one examination per pregnant woman and for obtaining homogeneity in the examinations, the Linear Integer Programming technique and LINGO 13 software (LINDO Systems Inc., Chicago, IL, USA) together with Microsoft Excel 2007 were used. The technique was executed a second time for the remaining examinations. The first sample was used to create the curve (training sample), and the second one was used to validate the model (test sample).

The protocol developed by Altman and Chitty^14^ was used to calculate the estimated fetal weight curves and correlate fetal weight (in grams) with gestational age (in weeks and days). One of the protocol's recommendations is that reference percentiles be calculated using cross-sectional studies, that is, with one observation per fetus. The standard deviation of the weight was modeled as a function of the gestational period. The quality of the model was evaluated by considering the coefficient of determination and analyzing the residues of the adjusted model. The model was estimated using the training dataset and validated in the second dataset. The generated model was compared graphically with the Hadlock model^13^ because the latter is the most commonly used. The generated model was also compared with the curve of the Intergrowth 21^st^ project,^15^ a prescriptive curve of how growth should occur under optimal conditions, which was designed for international use.

## Results

A sample of 2,211 ultrasound examinations was generated, one for each pregnant woman, of which 1,836 (83%) were white, 310 (14%) were mixed race, 44 (2%) were black, and 21 (1%) were Asian. There was adequate distribution across the 28 weeks of gestation analyzed (14 to 41 weeks of gestation) and a standard deviation of 0.173. We believe that the majority of the pregnant women had moderate/high socioeconomic status, because the ultrasound examinations were performed in a private clinic and all ultrasound examinations were private or covered by health insurance. The distribution of the examinations is presented in Table 1.

**Table 1:** Frequencies of cases at each gestational age

Gestational age (weeks)	Total ultrasound examinations	Examinations used to adjust the proposed model
14–14 + 6	128	46
15–15 + 6	226	58
16–16 + 6	295	76
17–17 + 6	323	82
1818 + 6	260	67
19–19 + 6	153	40
20–20 + 6	215	56
21–21 + 6	417	106
22–22 + 6	659	166
23–23 + 6	396	102
24–24 + 6	149	38
25–25 + 6	125	49
26–26 + 6	190	49
27–27 + 6	291	74
28–28 + 6	385	97
29–29 + 6	310	80
30–30 + 6	300	78
31–31 + 6	373	96
32–32 + 6	388	97
33–33 + 6	357	91
34–34 + 6	432	111
35–35 + 6	476	120
36–36 + 6	584	148
37–37 + 6	565	141
38–38 + 6	334	86
39–39 + 6	91	35
40–40 + 6	20	17
41–41 + 6	5	5
Total	8,447	2,211

The model was executed a 2^nd^ time using the remaining 6,236 examinations. This 2^nd^ execution used the same extraction method to generate a second sample with 1,957 examinations. The first sample was used to create the curve (training sample), whereas the second sample was used to validate the curve (test sample).

The gestational periods and fetal weights determined for the 2,211 examinations were considered for assessing the correlation between the gestational week and estimated fetal weight on ultrasound. The estimation of the model considered the gestational period in weeks as the explanatory variable and the natural logarithm of fetal weight on ultrasound as the response variable. The best fit was obtained using a quadratic model given by the following equation: . The estimated weight of each fetus according to the gestation period is given by: estimated weight = e^(0.6034575+0.3320483.week–0.003589055.week^2^)^.

The standard deviation of fetal weight was modeled according to the gestational period, as proposed by Altman and Chitty.^14^ For this estimation, the absolute values of the residues obtained with the model presented above were considered. The best ratio obtained for estimating the standard deviation was quadratic. Below is the model used for estimating the standard deviation of fetal weight as a function of the gestational week.



**Fig. 1:**
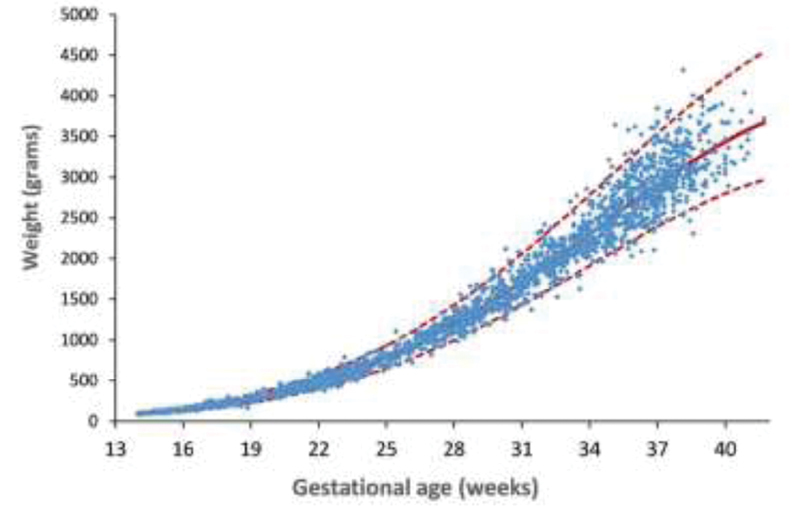
Fetal estimated weight and gestational ages of the evaluated cases. The adjusted curve correlating fetal weight and gestational period with a 95% confidence interval for the individual observations is also shown.

Therefore, the scores for each case were calculated using the following equation:



The model's coefficient of determination was R^2^= 99.11%, indicating that 99.11% of fetal weight variations were explained by the model. Fig. 1 shows the results of fetal weights and gestational periods of the evaluated cases. The adjusted curve correlating fetal weight and gestational period with a 95% confidence interval for the individual observations is also shown. The fetal weights in each gestational week for each percentile (3^rd^, 5^th^, 10^th^, 50^th^, 90^th^, 95^th^, and 97^th^) are shown in Table 2.

**Table 2:** Percentiles of estimated fetal weight (grams) at each gestational age

Gestational age (week)	3rd	5th	10th	25th	50th	75th	90th	95th	97th
14	78.8	80.6	83.5	88.5	94.5	100.9	107.0	110.8	113.4
15	99.2	101.4	105.0	111.3	118.7	126.6	134.2	138.9	142.1
16	123.9	126.7	131.2	138.9	148.0	157.8	167.1	172.9	176.8
17	153.7	157.2	162.6	172.1	183.3	195.2	206.6	213.8	218.5
18	189.3	193.4	200.1	211.7	225.3	239.8	253.7	262.4	268.2
19	231.3	236.4	244.4	258.4	275.0	292.6	309.4	319.9	326.9
20	280.6	286.7	296.4	313.3	333.2	354.4	374.7	387.3	395.8
21	337.8	345.2	356.8	377.0	400.9	426.3	450.5	465.6	475.7
22	403.8	412.5	426.3	450.4	478.9	509.1	537.9	555.9	567.9
23	479.0	489.4	505.7	534.3	567.9	603.6	637.7	659.0	673.3
24	564.1	576.3	595.5	629.1	668.7	710.7	750.8	775.9	792.6
25	659.5	673.7	696.2	735.5	781.7	830.9	877.8	907.1	926.6
26	765.2	781.8	807.9	853.6	907.3	964.5	1,019.0	1,053.1	1,075.8
27	881.4	900.5	930.7	983.5	1,045.6	1,111.6	1,174.7	1,214.1	1,240.4
28	1,007.8	1,029.7	1,064.4	1,124.9	1,196.3	1,272.2	1,344.6	1,389.9	1,420.1
29	1,143.7	1,168.7	1,208.3	1,277.5	1,358.9	1,445.6	1,528.4	1,580.1	1,614.7
30	1,288.5	1,316.8	1,361.7	1,440.2	1,532.7	1,631.1	1,725.0	1,783.8	1,823.1
31	1,440.9	1,472.8	1,523.4	1,611.9	1,716.2	1,827.3	1,933.4	1,999.8	2,044.2
32	1,599.4	1,635.2	1,691.9	1,791.0	1,908.0	2,032.6	2,151.7	2,226.3	2,276.2
33	1,762.3	1,802.1	1,865.2	1,975.7	2,106.1	2,245.0	2,378.0	2,461.3	2,516.9
34	1,927.4	1,971.5	2,041.3	2,163.6	2,308.0	2,462.1	2,609.6	2,702.0	2,763.8
35	2,092.5	2,141.0	2,217.8	2,352.3	2,511.3	2,681.1	2,843.7	2,945.7	3,013.9
36	2,255.0	2,307.9	2,391.8	2,538.9	2,712.9	2,898.9	3,077.1	3189.0	3,263.8
37	2,412.1	2,469.6	2,560.7	2,720.5	2,909.8	3,112.2	3,306.5	3,428.4	3,510.1
38	2,561.2	2,623.1	2,721.4	2,894.0	3,098.6	3,317.6	3,528.0	3,660.2	3,748.8
39	2,699.3	2,765.7	2,871.1	3,056.3	3,276.0	3,511.6	3,738.1	3,880.6	3,976.0
40	2,823.8	2,894.5	3,006.8	3,204.3	3,438.9	3,690.7	3,933.1	4,085.7	4,187.9

**Fig. 2:**
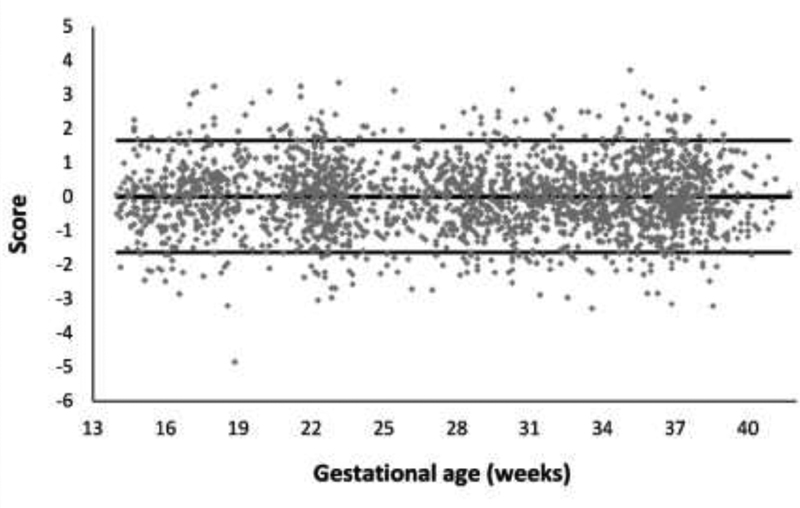
Calculated scores at a confidence interval of 90%.

Fig. 2 shows the calculated scores based on the above equation, together with the values of the standardized normal distribution (–1.64; 1.64), which indicates the range corresponding to 90% of the area under the curve of this distribution. For the model data, 89.5% of the scores were within this range, indicating a good adherence to the model. This percentage was 89% in the tested dataset, indicating a very good reproducibility of the model for estimating weights using data that were not used in the adjustment.

**Fig. 3:**
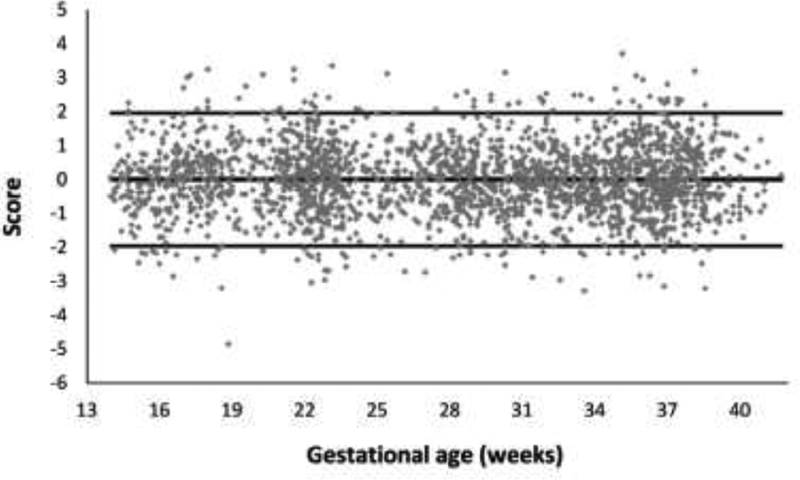
Calculated scores at a confidence interval of 95%.

Fig. 3 shows the calculated values based on the above equation together with the values of the standardized normal distribution (–1.96; 1.96), which indicate the range corresponding to 95% of the area under the curve of this distribution. For the model data, 94.1% of the scores were within this range, indicating good adherence to the model. This percentage was 94% in the tested dataset, indicating very good reproducibility of the model for estimating weights using data that were not used in the adjustment.

**Fig. 4:**
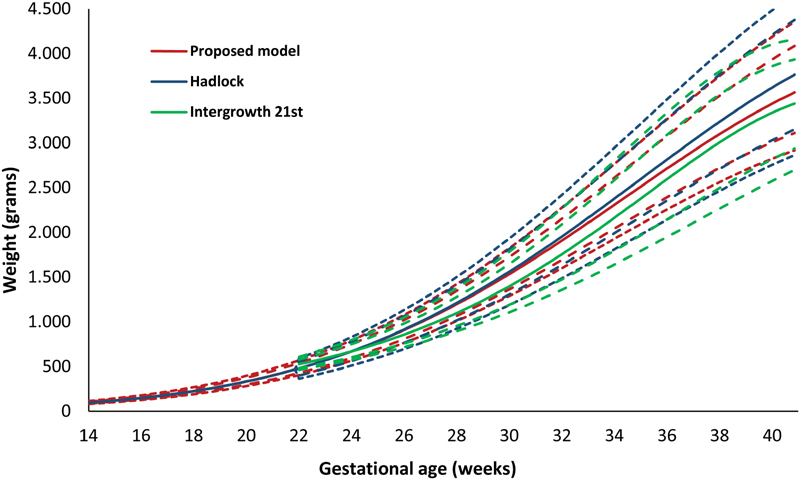
Proposed, Hadlock, and Intergrowth 21^st^ models according to fetal estimated weight—Curves for the 3^rd^, 10^th^, 50^th^, 90^th^ and 97^th^ percentiles.

The proposed model was compared graphically with the Hadlock references and the curves developed in the Intergrowth 21^st^ project (Fig. 4).

**Fig. 5:**
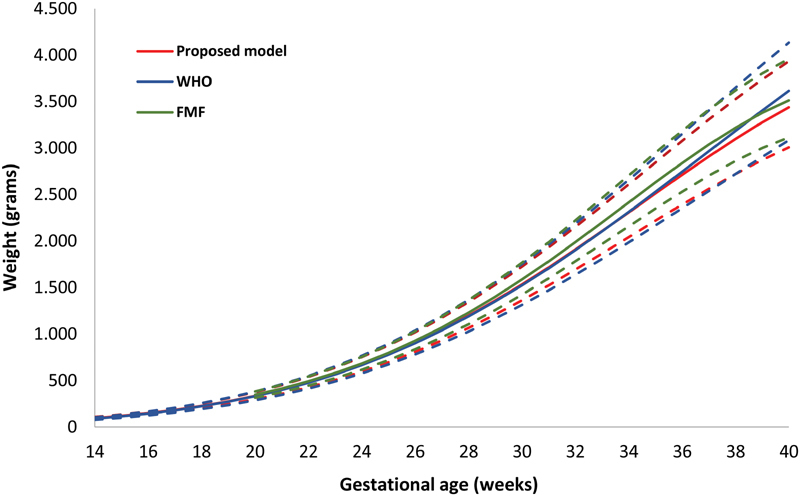
Proposed, Fetal Medicine Foundation, and World Health Organization models according to fetal estimated weight—Curves for the 10^th^, 50^th^, 90^th^ percentiles.

Additionally, the proposed model was compared graphically with the Fetal Medicine Foundation (FMF),^16^ and the World Health Organization (WHO),^17^ models (Fig. 5).

## Discussion

We have developed reference curves for estimated fetal weight for a local population based on the hypothesis that these curves were different from those established for other populations. The distribution of racial population was in agreement with the data from the last local census,^12^ despite being a population with moderate/high socioeconomic status from a private service. The proposed model was constructed after excluding gestations at risk of growth disorders and by including normal fetuses, thus defining a reference curve.

Following the recommendation of Altman and Chitty,^14^ each fetus was included only once because the inclusion of multiple observations of the same fetus would characterize a growth curve, and, in this case, the effective sample size tends to be the number of fetuses and not the number of observations. Although our data were collected retrospectively, the use of Linear Integer Programming allowed us to select only one observation for each fetus, with homogeneous distribution across the evaluated gestational weeks. The use of this technique also allowed evaluating the reproducibility of the model in a population that was not included in the study, and the results indicated that reproducibility was very good in this population. Another advantage of the selected sample is that all patients had first trimester ultrasonography data for adequate determination of gestational age.

A disadvantage of the retrospective nature of the study is that it did not allow confirming whether a factor that could affect fetal growth was truly absent. Another limitation was that there was no standardization of data collection or blinding of the measurements on the screen of the ultrasound devices for the examiner, and this limitation might interfere with the obtained values. Moreover, the obtained curves might be affected by the fact that some examinations might have been requested by clinical indication because of suspected pathological growth. However, we believe that the influence of this factor, if present, is minimal because an overrepresentation of fetuses that were small for gestational age (SGA) and/or with IUGR or large for gestational age (LGA) in this sample would result in lower percentile values at the lower limit and an increase in curve percentiles, respectively, in contrast with what we observed by comparing our curves with other reference curves.

Compared with the Hadlock curves,^13^ the 50^th^, 90^th^, and 97^th^ percentiles in the proposed model were lower than those found by Hadlock, whereas the 10^th^ percentile almost overlapped, and the 3^rd^ percentile was slightly higher in our model. With regard to the curves of Intergrowth 21^st 15^ the 3^rd^, 10^th^, and 50^th^ percentiles were higher in our model. Additionally, the proposed model was also compared with the Fetal medicine Foundation (FMF)^16^ and World health Organization (WHO)^17^ curves showing slight differences. While the 10^th^ percentile was similar for the proposed and the WHO models, the FMF model was higher. With regarding to the 90^th^ percentile, the proposed model was lower. Therefore, the use of all five models in the same population would result in different fetal classifications for estimated fetal weight and could reflect differences in the populations used for constructing the references.

Two Brazilian studies developed estimated fetal weight curves based on local populations^18^ ^19^ in the cities of São Paulo and Campinas, which are both located in the southeast of Brazil, and both found that the mean values were slightly lower than those presented by Hadlock et al.^13^ A similar result was also observed in the model proposed in the current study for higher percentiles, but it was not observed in the 3^rd^ and 10^th^ percentiles, demonstrating that local variations might occur within a single country. Addressing these small differences may be important for adequately diagnosing growth disorders. Therefore, evaluating the performance of these curves in the populations for which they were developed is essential to assess whether they are in fact more adequate than the curves currently in use.

## Conclusion

In summary, we provide reference curves for estimated fetal weight for a local population living in the city of Curitiba, South of Brazil. The differences relative to the Hadlock and Intergrowth 21st Project curves may reflect differences in the populations on which the models were based. The implications of these differences for prenatal management and perinatal outcome could not be assessed in this study, and further research is needed to assess the application of these models to local populations for determining such implications.
